# Three case reports of pulmonary mucormycosis with a review of the literature

**DOI:** 10.3389/fmed.2025.1580912

**Published:** 2025-07-09

**Authors:** Zhengyang Zhang, Min Wang

**Affiliations:** ^1^Shandong University of Traditional Chinese Medicine, Jinan, China; ^2^The Second Affiliated Hospital of Shandong University of Traditional Chinese Medicine, Jinan, China

**Keywords:** pulmonary mucormycosis, case report, literature review, amphotericin B, bronchoalveolar lavage

## Abstract

Pulmonary mucormycosis (PM) is an invasive and life-threatening fungal infection that predominantly affects immunocompromised individuals. This study thoroughly examined the disease through three case reports and a literature review. Case 1 involved a patient with type 1 diabetes mellitus diagnosed through bronchoscopic histopathology, who succumbed despite a combination of oral isavuconazole, nebulized amphotericin B, and intravenous amphotericin B cholesteryl sulfate complex. Case 2 involved a patient with follicular non-Hodgkin lymphoma who had a concurrent coronavirus disease 2019 (COVID-19) infection, which was confirmed through metagenomic next-generation sequencing (mNGS) of bronchoalveolar lavage fluid (BALF). The patient experienced clinical improvement following sequential intravenous voriconazole, amphotericin B cholesteryl sulfate complex, and oral isavuconazole. Case 3 involved a patient diagnosed with mNGS in a lung cancer patient with chronic obstructive pulmonary disease, who showed poor therapeutic response to combined intravenous voriconazole, amphotericin B cholesteryl sulfate complex, and oral isavuconazole, resulting in fatal outcomes. Literature synthesis revealed mortality rates of 28.3% with antifungal monotherapy compared to 23.7% when antifungal monotherapy was combined with bronchoscopic intervention; the mortality rate for antifungal–surgical combination therapy was 9%. Notably, all 13 patients receiving multimodal treatment (antifungals, bronchoscopy, and surgery) survived. These findings underscore that combination therapy integrating pharmacotherapy, bronchoscopic intervention, and surgical resection demonstrated significantly superior survival outcomes compared to monotherapy.

## 1 Introduction

Mucormycosis associated with COVID-19 gained attention late in the pandemic when thousands of cases were reported in India, the diabetes capital of the world. Most human infections with mucormycosis are caused by *Rhizopus*, *Pedicellaria*, *Mucor*, and *Rhizopus*. Still, there are other clinically relevant organisms in the Mucoridales, including *Actinomyces*, *Apophysomyces*, *Cunninghamella*, *Lichtheimia* (formerly known as *Absidia*), *Saksenaea*, and *Syncephalastrum* (1). Pulmonary mucormycosis (PM) is an infection that occurs in the lungs and is the third most common site of infection after the rhino-orbital-cerebral (ROC) region and the skin. PM occurs predominantly in patients with compromised immune systems. Risk factors for PM include hematological malignancies, solid organ transplantation, diabetes, and diabetic ketoacidosis (2). Uncontrolled diabetes, coronavirus disease 2019 (COVID-19)-associated hypoxia, and subsequent glucocorticoid use were found to be independent risk factors for pulmonary mucormycosis and were associated with a very high mortality rate (3). Here, we report three confirmed cases of pulmonary mucormycosis, along with complete clinical data, and then searched the PubMed database using “pulmonary mucormycosis” as a key search term to screen the English-language publications from 2022 to 2024 for cases of pulmonary mucormycosis infection that met the criteria and perform literature analysis. The results were used to investigate the clinical features, imaging characteristics, and diagnosis and treatment methods of pulmonary mucormycosis, thereby improve the understanding of the disease.

## 2 Case presentation

### 2.1 Case 1

**Figure 1:**
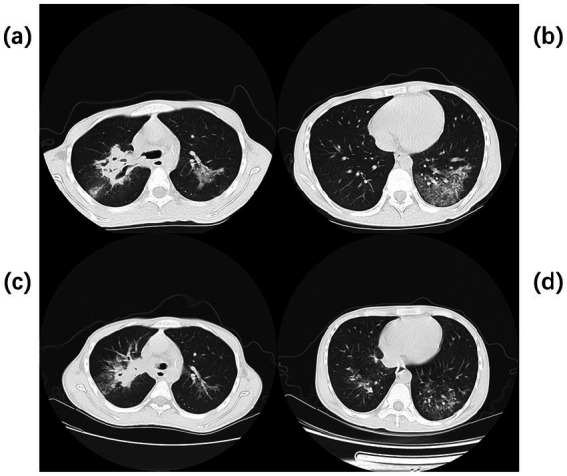
Chest CT of Case 1. Computed tomography (CT) of the chest before the start of treatment **(a,b)** Multiple flaky, glassy, nodular hyperdense shadows in both lungs, partly solid, especially in the upper lobe of the right lung, with cavity formation. Post-treatment chest CT **(c,d)** The inflammation is more absorbed than before, but cavities remain.

**Figure 2:**
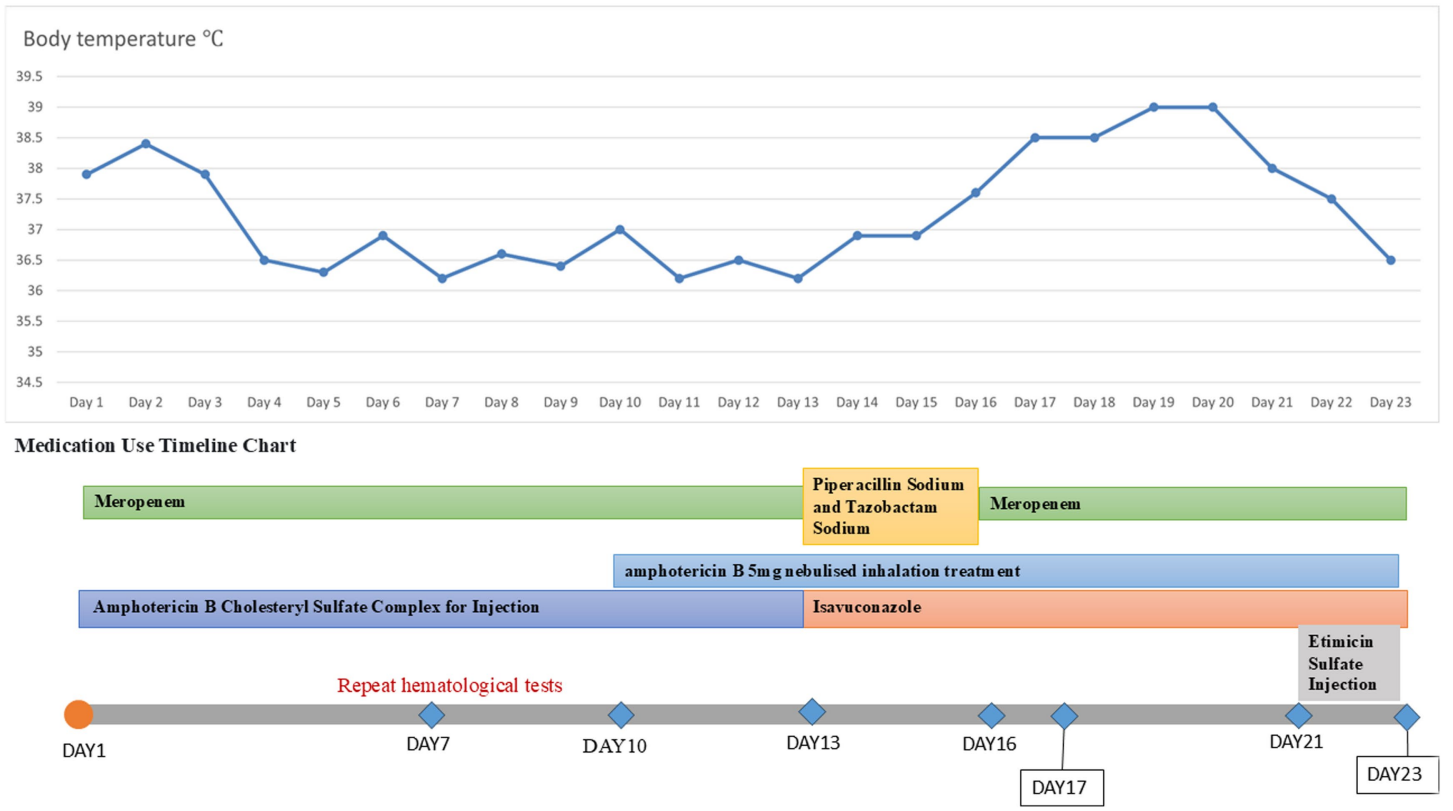
Temperature chart and medication chart for Case 1.

A 20-year-old male was admitted to the hospital on 28 February 2024 for treatment of cough and sputum with wheezing for more than 1 month. He had a history of type I diabetes mellitus for many years with poor glycemic control. He had undergone fiberoptic bronchoscopy at another hospital, where lung tissue was forceps extracted, and the pathology results showed “fungal disease of the upper lobe of the right lung and the middle segment of the right lung, with a morphology consistent with a mucormycosis infection.” The patient was referred to our hospital for further diagnosis and treatment. Upon admission, the physical examination revealed the following: body temperature, 37.9°C; pulse rate, 96 beats per minute; respiratory rate, 24 breaths per minute; blood pressure, 130/78 mm Hg; and oxygen saturation, 94%. The patient presented with an extremely emaciated habitus, mild dyspnea, coarse breath sounds bilaterally, and decreased breath sounds over the right lung fields. Laboratory investigations revealed the following: A complete blood count demonstrated a white blood cell count of 4.60 × 10^9^/L (reference range: 4.0–10.0 × 10^9^/L) with a neutrophil percentage of 75.5% (reference range: 50–70%), within the normal leukocyte range but with a mildly elevated neutrophil proportion. C-reactive protein was 39.9 mg/L (reference range: <10 mg/L), indicating a significantly elevated level, indicating an active inflammatory process. Serum albumin measured 29.2 g/L (reference range: 35–50 g/L), consistent with hypoalbuminemia. Severe acute respiratory syndrome coronavirus 2 (SARS-CoV-2) was negative (detected by polymerase chain reaction). Sputum culture demonstrated *Klebsiella pneumoniae* positive for extended-spectrum *β*-lactamase (ESBL). Chest computed tomography (CT): multiple flaky, ground-glass, and nodular high-density shadows are visible in both lungs, with some solid areas; the upper lobe of the right lung is prominent, and cavity formation can be seen (Figure 1). The patient was admitted with intermittent fever (peak temperature, 38.4°C), cough, and expectoration, receiving symptomatic supportive treatment including meropenem 1 g every 8 h for antimicrobial therapy, insulin for glycemic control, correction of electrolyte disorders, fluid resuscitation, and oxygen therapy. Pathological diagnosis confirmed pulmonary mucormycosis, and intravenous amphotericin B cholesteryl sulfate complex was initiated at 50 mg (gradually titrated to 150 mg) for antifungal treatment. At admission, the fasting blood glucose level was 22.48 mmol/L, which was managed with 10 units of insulin administered thrice a day and 10 units of long-acting insulin administered once nightly. Due to inadequate glycemic control, treatment was switched to an insulin pump (basal rate 124 units/24 h and preprandial bolus 12 units). The patient’s condition stabilized 4 days after admission, without recurrence of fever. On the seventh day of admission, repeated blood tests showed white blood cell count 6.38 × 10^9^/L with neutrophil percentage 62.1%; C-reactive protein 9.6 mg/L; and serum albumin 34.7 g/L. On day 10 of admission, amphotericin B nebulization was added to the combined antifungal therapy against mucormycosis. On the 13th day of admission, a chest CT scan of the right lung lesion revealed improved density, with parts of the lesion being density lower in density than in the previous scan, and the range was reduced (Figure 1). Meropenem was de-escalated to piperacillin sodium and tazobactam sodium. Amphotericin B cholesteryl sulfate complex for injection was discontinued after being administered at full dose and course for 10 days, and switched to isavuconazole 200 mg once daily. On the 16th day of admission, the patient developed a fever again, with a fever peak of 39.8°C. Repeat hematological tests showed the following: White blood cell count, 8.91 × 10^9^/L; neutrophil percentage, 83.4%; and C-reactive protein, 48.1 mg/L. Considering the suspicion of recurrent pulmonary mucormycosis and the patient’s extremely emaciated condition, combined with the angioinvasive nature of mucormycosis, bronchoscopy was deemed to carry a high risk of massive airway hemorrhage due to mucosal fragility. The risk–benefit ratio of the invasive procedure was thoroughly explained to the family, who declined bronchoscopy after complete counseling. Antibiotic therapy was re-escalated to meropenem. On day 21 of admission, a review of sputum culture results confirmed *K. pneumoniae*. Combination antimicrobial therapy with intravenous meropenem and etimicin sulfate was initiated, but demonstrated suboptimal response, with body temperature persistently above 38.0°C. On day 23 of admission, the family requested discharge, and cefdinir was prescribed for use during transfer. Two months into follow-up, it was found that the patient had died. Temperature chart and medication chart for Case 1 (Figure 2).

### 2.2 Case 2

**Figure 3:**
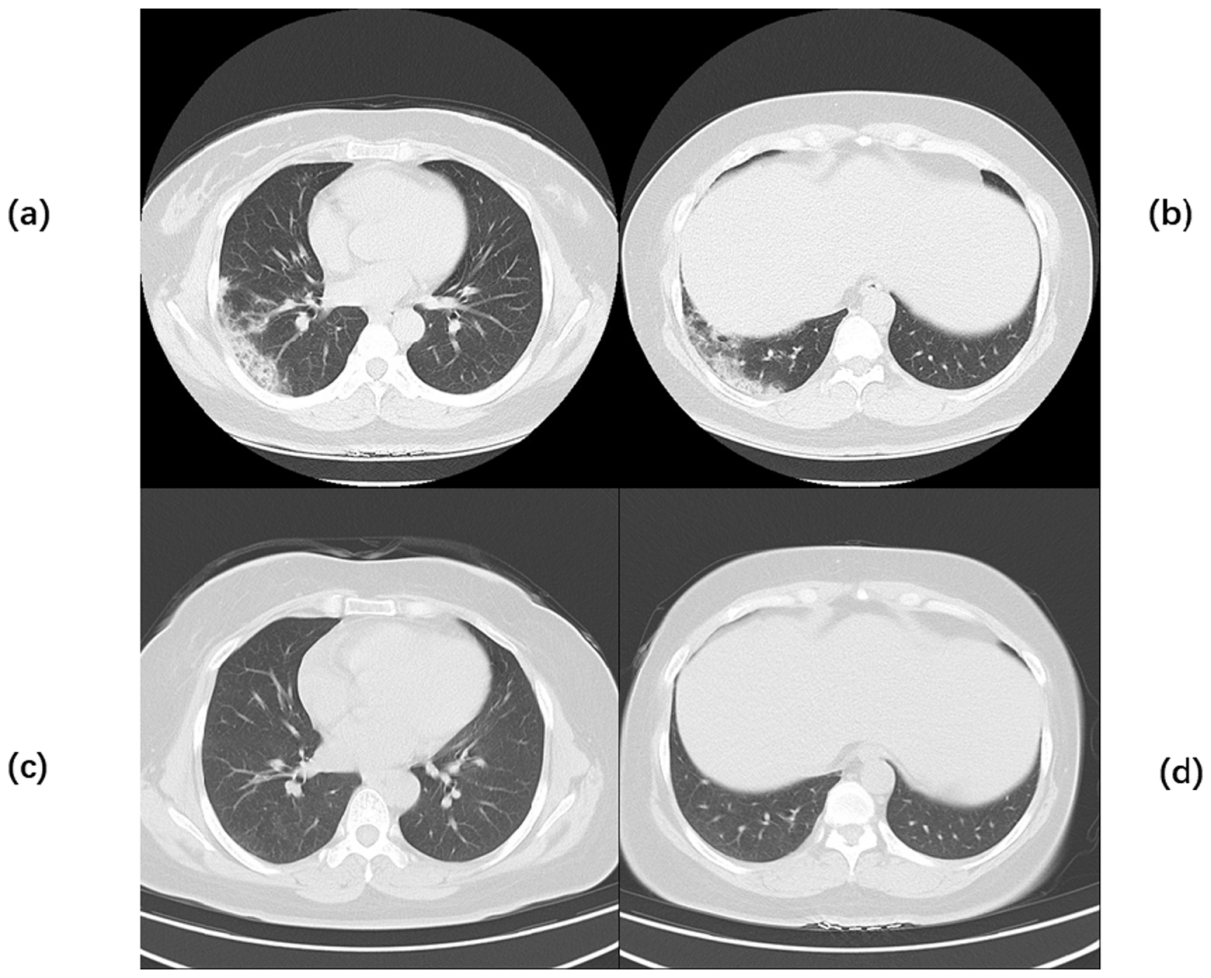
Chest CT of Case 2. Computed tomography (CT) of the chest before the start of treatment **(a,b)** large, patchy high-density foci under the pleura of the right lung with blurred margins and uneven density. Chest CT after 2 months of continuous treatment **(c,d)** Flocculent and patchy slightly high-density foci were seen under the pleura of the lower lobe of the right lung, with fuzzy edges and uneven densities, which were significantly better than before.

**Figure 4:**
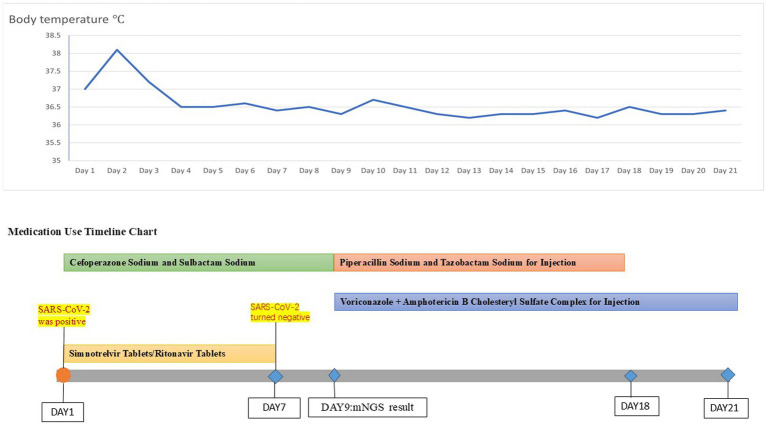
Temperature chart and medication chart for Case 2.

A 42-year-old woman was admitted to the hospital on 19 September 2023, with a 2-month history of paroxysmal cough and wheezing. She had a 2-year history of follicular non-Hodgkin lymphoma and had received maintenance therapy with Obinutuzumab. It is worth mentioning that the patient was admitted to an outside hospital on several occasions for coughing, wheezing, expectoration of white mucoid sputum, fever up to 38°C, and generalized fatigue. At the outside hospital, a chest CT showed left lung pneumonia, and she received symptomatic treatment with moxifloxacin, methylprednisolone, and bromhexine. Two days after hospital discharge, her symptoms worsened, and she was admitted to our hospital for further management. Upon admission, physical and laboratory examinations were performed. Physical examination revealed temperature, 37°C; pulse rate, 78 bpm; respiratory rate, 19 rpm; blood pressure, 125/78 mm Hg; oxygen saturation, 97%. Bilateral coarse breath sounds with scattered wet rales were auscultated. Laboratory investigations showed: complete blood count demonstrated white blood cell count of 4.21 × 10^9^/L (normal range: 4.0–10.0 × 10^9^/L), eosinophil count of 0.03 × 10^9^/L (normal range: 0.05–0.5 × 10^9^/L). C-reactive protein was 84.0 mg/L (normal range: <10 mg/L), significantly elevated, indicating active inflammation. Chest CT showed large, patchy high-density foci in the subpleural regions of the right lung with blurred margins and uneven density. The patient was admitted with fever (peak 38.1°C), cough with sputum production, and a positive SARS-CoV-2 nucleic acid test (polymerase chain reaction (PCR)). Simnotrelvir tablets/Ritonavir tablets (co-packaged) were initiated, along with intravenous Cefoperazone sodium/Sulbactam sodium for antibacterial treatment and ambroxol hydrochloride for symptomatic relief to facilitate sputum expectoration. On the seventh day of admission, the patient’s SARS-CoV-2 test (PCR) result turned negative; however, the patient continued to present with cough and sputum production. Fiberoptic bronchoscopy was performed to clarify the underlying etiology, and bronchoalveolar lavage fluid (BALF) was sent for metagenomic next-generation sequencing (mNGS) and culture. On the ninth day of admission, the results showed the presence of *K. pneumoniae*, *Rhizopus delemar*, *Candida albicans*, *Rhizopus oryzae*, and parainfluenza virus. Where *R. delemar* (reads 710 signal intensity: high), *R. oryzae* (reads 453 signal intensity: high). Both are Mucoraceae fungi. Culture of BALF revealed *Aspergillus flavus*. Intravenous piperacillin-tazobactam was administered to combat the bacterial infection. Voriconazole (0.3 g every 12 h) was administered for the treatment of Aspergillus infection. Amphotericin B cholesteryl sulfate complex for injection (50 mg, gradually titrated to 250 mg) was initiated as antifungal therapy against mucormycosis. On the 17th day of admission, repeat blood tests revealed: white blood cell count 3.19 × 10^9^/L (normal range: 4.0–10.0 × 10^9^/L); monocyte percentage 15.5% (normal range: 3–10%); lymphocyte count 0.44 × 10^9^/L (normal range: 0.8–4.0 × 10^9^/L); and serum albumin concentration 36.7 g/L (normal range: 35–50 g/L). After 21 days of hospitalization and sequential antifungal therapy with Isavuconazole, the patient was discharged. Two months after continuous therapy, a repeat chest CT revealed patchy and flocculent foci with mildly increased density in the subpleural regions of the right lower lobe, characterized by blurred margins and heterogeneous attenuation. These findings were significantly improved compared to prior scans (Figure 3). Temperature chart and medication chart for Case 2 (Figure 4).

### 2.3 Case 3

**Figure 5:**
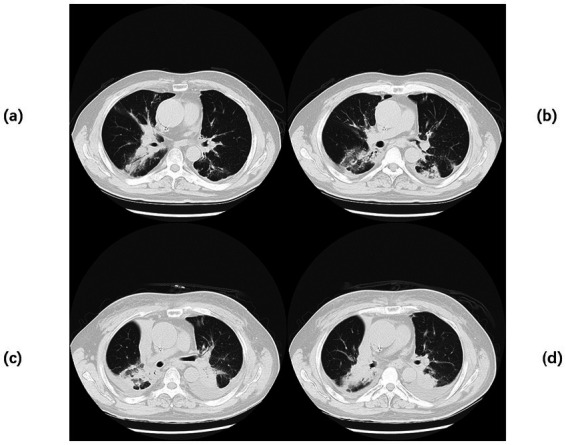
Chest CT of Case 3. Computed tomography (CT) of the chest before the start of treatment **(a,b)** Multiple scattered patchy high-density lesions were seen in both lungs, some of which were ill-defined and mostly solid, and bronchial air phases were seen within some of the lesions. Chest CT after exacerbation **(c,d)** A small amount of pericardial effusion was seen, and arcuate watery density shadows were seen in both thoracic cavities.

**Figure 6:**
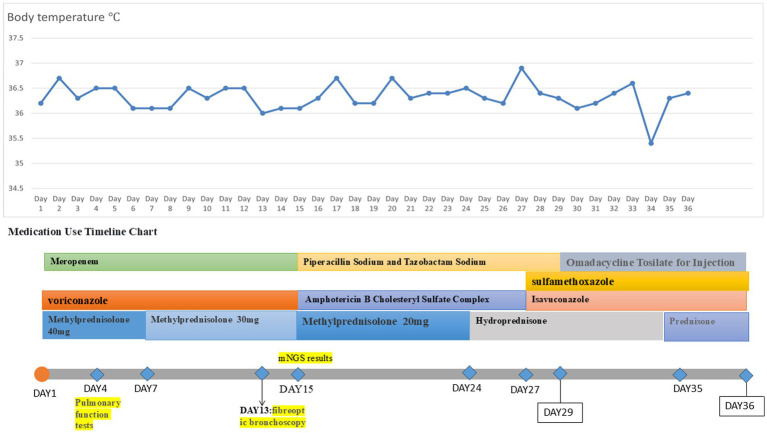
Temperature chart and medication chart for Case 3.

A 57-year-old male patient was admitted with a 3-month history of cough and sputum production. He had a 2-year history of squamous cell esophageal cancer and lung squamous cell carcinoma, having undergone multiple courses of radiotherapy and immunotherapy. Sputum culture at an outside hospital previously revealed *Aspergillus* species, and oral voriconazole was administered, resulting in a suboptimal response. He also had a 2-year history of hypothyroidism. The patient was admitted to our hospital with cough, expectoration of copious yellowish-white mucoid sputum, and dyspneic wheezing, with symptoms worsening on exertion. Upon admission, the patient underwent physical examination, laboratory testing, and ancillary diagnostic evaluations. Physical examination revealed the following: Temperature, 36.2°C; pulse rate, 82 beats per minute; respiratory rate, 20 breaths per minute; blood pressure, 124/78 mm Hg; and oxygen saturation, 93%. Coarse breath sounds were noted bilaterally, with scattered rales detected on auscultation. Laboratory investigations showed: complete blood count demonstrated a white blood cell count of 7.19 × 10^9^/L (normal range: 4.0–10.0 × 10^9^/L), neutrophil percentage of 93.9% (normal range: 50–70%), and C-reactive protein concentration of 19.0 mg/L (normal range: <10 mg/L). Ancillary investigations revealed chest CT findings: multiple scattered, patchy, hyperdense foci in both lungs, some with ill-defined margins and predominantly solid opacity, others demonstrating air bronchograms within. Upon admission, the patient received symptomatic management including meropenem for anti-infective therapy, voriconazole for antifungal treatment, and methylprednisolone 40 mg to alleviate inflammation. On the fourth day of admission, pulmonary function tests were performed: very severe mixed ventilation dysfunction, predominantly obstructive (forced expiratory volume in 1 s (FEV1)/forced vital capacity (FVC) 46.86%; percentage predicted FEV1 (FEV1%pred) 33%, FEV1/FVC 51.55%, and FEV1pred 34% after inhalation of bronchodilators), and the additional diagnosis: chronic obstructive pulmonary disease. Inhalation treatment with Breztri Aerosphere. On day 13 of admission, the patient underwent fiberoptic bronchoscopy, and bronchoalveolar lavage fluid (BALF) was sent for metagenomic next-generation sequencing (mNGS) and culture. On day 15 of admission, results showed: see *K. pneumoniae* (reads 2,961 signal strength: strong), *Rhizopus arrhizus* (reads 101 signal strength: medium). Due to suboptimal response to voriconazole, treatment was promptly switched to intravenous amphotericin B cholesteryl sulfate complex, initiated at 50 mg daily and gradually titrated to 250 mg daily as primary antifungal therapy. After 10 days of full-dose ABCD, the regimen was transitioned to oral isavuconazole for antifungal maintenance therapy. On the 25th day of admission, the patient had oedema of the face and neck. Chest CT + pulmonary artery computed tomography angiography (CTA): CT showed: scattered multiple patchy foci of increased density in both lungs, some with unclear borders, most of them solid, some with bronchial air phases, and some with thickened interlobular septa. Multiple nodules were seen in both lungs, the larger one being a solid nodule in the dorsal segment of the lower lobe of the left lung. A small amount of pericardial effusion was observed, and arcuate watery density shadows were seen in both thoracic cavities. CTA of the pulmonary artery did not show any significant abnormality (Figure 5). Ultrasound of the upper limb suggests extensive thrombosis of the left upper limb vein. On day 27 of admission, laboratory investigations revealed elevated C-reactive protein at 57.1 mg/L (normal <10 mg/L), brain natriuretic peptide at 246 pg./mL (normal <100 pg./mL), and D-dimer at 1.80 mg/L (normal <0.5 mg/L). Current imaging demonstrated progression from prior studies, with concomitant upper limb venous thrombosis. There was a risk of thrombus extension or embolic dislodgement. For anticoagulation, thrombolysis was considered; however, due to the risk of fatal hemorrhage (e.g., intracranial hemorrhage), the family declined this approach after thorough discussion. Anticoagulation therapy was subsequently intensified. The patient’s condition suddenly worsened on the 29th and 34th days after admission, respectively, and the patient was resuscitated, which was ultimately successful. On the 36th day after admission, the patient’s family was again informed of the patient’s condition. The patient currently has multiple coexisting pulmonary diseases (lung cancer, radiation pneumonitis, secondary infections, and COPD), with recurrent episodes of shortness of breath and progression of previous conditions. Recurrent thromboembolic events further complicated the case, and the prognosis remained poor. The family members indicated their understanding and decided to transfer the patient to the intensive care unit (ICU) of a tertiary hospital in Beijing, China, for further treatment. We had conducted telephone communication with the hospital, but details regarding subsequent treatment remained unclear. During a telephone follow-up 1 month later, we were informed that the patient had passed away. Temperature chart and medication chart for Case 3 (Figure 6).

## 3 Literature review

A comprehensive literature search was conducted in the PubMed database using the keywords “pulmonary mucormycosis” and “mucormycosis” to identify English-language case reports published between 2022 and 2024. Sixty cases meeting inclusion criteria were extracted, with three additional cases from this study incorporated, yielding a total cohort of 63 patients (detailed data in Table 1). Among the 63 pulmonary mucormycosis cases, 42 were men (66.7%), 20 were women (31.7%), and one case lacked documentation of gender. The median age was 52 years [interquartile range (IQR), 34–60 years]. Treatment outcomes were available for 61 cases, demonstrating clinical improvement in 42 (68.9%) and mortality in 19 (31.1%). The most prevalent underlying comorbidities included diabetes mellitus (DM), encompassing diabetic ketoacidosis (DKA) and diabetic nephropathy (*n* = 32, 50.8%), COVID-19 infection (*n* = 17, 27.0%), hematological malignancies (*n* = 6, 9.5%), and post-renal transplantation (*n* = 4, 6.3%). Other comorbidities comprised traumatic injuries (*n* = 2, 3.2%), substance abuse, HIV infection, and systemic lupus erythematosus (*n* = 1 each, 1.6%). Thoracic CT manifestations included reversed halo sign (*n* = 2, 3.2%), air crescent sign (*n* = 1, 1.6%), mass/consolidation (*n* = 29, 46.0%), and cavitation (*n* = 9, 14.3%). Diagnostic confirmation utilized bronchoalveolar lavage fluid (*n* = 25, 39.7%), tissue biopsy (*n* = 23, 36.5%), postoperative pathology (*n* = 13, 20.6%), and sputum/blood/pleural fluid (*n* = 11, 17.5%), with metagenomic next-generation sequencing (mNGS) employed in 36% of specimens. Antifungal therapy was administered to 53 patients, with mortality observed in 15 cases (28.3%), clinical improvement in 37 cases (69.8%), and an undocumented prognosis in 1 case. Among 38 patients receiving combined antifungal therapy and bronchoscopic intervention, mortality decreased to 23.7%. Twenty-eight patients demonstrated improvement (73.7%), and the outcome of one patient was not documented. Antifungal–surgical combination therapy yielded superior outcomes, with a 9% mortality and a 91% clinical improvement. Notably, all 13 patients undergoing multimodal treatment (antifungals, bronchoscopy, and surgery) survived (Table 2).

**Table 1:** Detailed information on the 60 case reports screened.

Case	Age	Sex	Concomitant disease	Disease duration	Chest CT	Diagnostic specimen	Therapeutic drug	Bronchoscopic therapy	Surgery	Outcome
1 (14)	20	M	AP + DKA	20 days	Atelectasis	BALF mNGS	AmB + Isavuconazole	Yes	No	Death
2 (15)	63	M	HF; Hypertension; DM; Post-renal transplantation; COVID-19	NM	Mass-like consolidation with cavity	BALF mNGS+ biopsy	Isavuconazole	Yes	cuneiform resection	Recover
3 (15)	60	W	ALL	NM	Mass	Biopsy	AmB + Isavuconazole	Yes	total lung resection	Recover
4 (16)	25	M	T-ALL	NM	Mass	Blood +BALF mNGS	AmB + PCZ	Yes	thoracoscopic surgery	Recover
5 (17)	11	M	AA	45 days	RHS	Pleural effusion mNGS	L-AmB, Caspofungin + Isavuconazole	No	left lower lobectomy	Death
6 (18)	54	W	DM	15 days	Abscess	BALF mNGS+ biopsy	AmB + Isavuconazole	Yes	No	Recover
7 (19)	60	M	DM	66 days	Crescent sign + hypodense	BALF mNGS + Pathology	L-AmB + VCZ	Yes	cuneiform resection	Recover
8 (20)	55	M	Hypertension, DM	NM	Cystic cavity+ Peripheral consolidation	Biopsy	NM	Yes	No	Death
9 (12)	37	W	DKA	12 days	Consolidation	Biopsy +BALF+EBUS-TBNA	AmB + PCZ	Yes	No	Recover
10 (21)	18	M	DKA	30 days	Consolidation	Biopsy +BALF+EBUS-TBNA	L-AmB	Yes	No	Recover
11 (22)	27	M	Post-renal transplantation +DM	NM	Circular pneumonia	Biopsy	L-AmB + PCZ	No	Yes	Recover
12 (23)	60	W	SARS-CoV-2	62 days	Mass	Biopsy	L-AmB + Isavuconazole + Methylprednisolone	Yes	No	Death
13 (24)	70	M	Hypertension AML Fractured neck of right femur	103 days	Thick-walled cavity	Hydrothorax mNGS	AmB + Isavuconazole	Yes	No	Recover
14 (25)	45	W	DKA	4 months	Tubercular shadow	BALF mNGS	AmB + PCZ	Yes	No	Recover
15 (26)	17	M	DM + COVID-19	NM	RHS	Biopsy	NM	NM	NM	NM
16 (27)	34	M	Epilepsy +DM	26 days	High density shading	BALF mNGS+ Biopsy	ABLC	Yes	No	Recover
17 (28)	65	M	Myelodysplastic syndrome	NM	Lump-like consolidation	Biopsy	AmB	No	No	Death
18 (29)	60	M	DM	NM	Airway wall thickening	Pathological tissue mNGS	AmB + PCZ	Yes	No	Recover
19 (30)	67	NM	ALL	50 days	Consolidation	sputum culture	L-AmB + Isavuconazole	No	No	Recover
20 (31)	8	W	SLE + Lupus nephritis	NM	Consolidation	BALF mNGS	AmB	Yes	local open drainage	Recover
21 (32)	73	W	DM	NM	Ground glass opacity	BALF mNGS + Biopsy	L-AmB + Itraconazole	Yes	No	Recover
22 (33)	75	M	Hypertension + DM + SARS-CoV-2	11 days	Consolidation	endotracheal aspirate culture	L-AmB	No	No	Death
23 (33)	60	M	Hypertension + DM + SARS-CoV-2	32 days	NM	BALF mNGS	L-AmB	Yes	No	Death
24 (34)	No	M	DM	NM	Mass	BALF mNGS	AmB + AmBD+ Isavuconazole	Yes	Lobectomy of the right lower lobe	Recover
25 (35)	50	M	Hypertension + DM + Gouty arthritis	9 days	High density lesion	BALF mNGS	AmBD	Yes	No	Death
26 (36)	60	W	DM + Breast Cancer	NM	Cavitation+ mass	Bronchial secretion PCR assay	L-AmB	Yes	No	Recover
27 (37)	41	M	COVID-19 + DM	NM	Pseudoaneurysm of the right lower lobe pulmonary artery	physiology	L-AmB	Yes	open heart surgery	Recover
28 (38)	52	W	DM	9 days	Bilateral lower lobe infected lesions	BALF mNGS+ physiology	NM	Yes	No	Death
29 (39)	56	M	HIV + ChronicHepatitis C + COPD	NM	Hyperintense lesions	Biopsy	Isavuconazole +PCZ	Yes	No	Death
30 (40)	43	M	NO	NM	Soft-tissue swelling	physiology	NM	No	left lower lobectomy	Recover
31 (41)	35	M	DKA	NM	Total collapse of the left lung	Biopsy	L-AmB + PCZ	Yes	total lung resection	Recover
32 (42)	16	M	Take drugs	NM	Pleural effusion and effusion in the right lung leading to a collapsed solid lesion	physiology	PCZ	No	thoracoscopic operation	Recover
33 (43)	65	M	NO	79 days	Bilateral gross glass shadows and solid lesions in the lower lobes of the lungs	Biopsy	Linezolid + Clarithromycin Tablet + AmB	Yes	No	Death
34 (44)	52	W	DM + hypothyroidism	28 days	Hygroma	physiology	L-AmB	No	Lower left lung cystectomy	Recover
35 (13)	56	M	NO	230 days	severe pneumonia	BALF culture	L-AmB + VCZ + PCZ	Yes	No	Recover
36 (13)	34	M	NO	346 days	Cavitary lesion	BALF culture	PCZ + AmBD	Yes	No	Recover
37 (45)	3	W	B-ALL	104 days	Nodular lesion	Blood and sputum mNGS	AmB + PCZ	No	Yes	Recover
38 (46)	6	M	chronic granulomatous disease	7 days	Pleural effusion	Biopsy	L-AmB	No	No	Recover
39 (47)	45	M	NO	56 days	Mass	NM	PCZ + AmB	Yes	No	Recover
40 (48)	31	W	COVID-19 ARDS+ pregnancy	169 days	Multi-atrial cavity with multifocal solid lesions	BALF culture	PCZ	Yes	Yes	Recover
41 (49)	34	W	HIV + hypertension +DM + asthma + Chronic Pancreatitis	NM	Solid lesion of the right middle and lower lungs	Biopsy	AmB + Isavuconazole	No	Yes	Death
42 (50)	69	M	COVID-19	170 days	Showing multiple cavities with low attenuation of real variables	BALF+ Biopsy	VCZ + L-AmB	Yes	No	Death
43 (51)	46	M	Renal transplant	NM	Solid change in the right lower lobe	BALF+ Biopsy	AmB + PCZ	Yes	open heart surgery	Recover
44 (52)	61	M	hypertension + SARS-CoV-2	109 days	Turbid nodular	physiology	L-AmB + Isavuconazole	No	Right Total Lung Resection	Recover
45 (53)	49	M	DKA + B-type influenza	NM	Pleural effusion	BALF	Isavuconazole	Yes	No	NM
46 (54)	63	M	Renal transplant + COVID-19	13 days	Pleural effusion + solid changes	Pleural fluid culture	L-AmB	No	No	Death
47 (55)	61	M	Diabetes mellitus with nephropathy	39 days	Cavity nodules + progressive solid changes	BALF+ Biopsy	VCZ + L-AmB + PCZ	Yes	No	Recover
48 (56)	40	M	DM	26 days	Useless weakling	EBUS-TBNA	AmB + PCZ	Yes	right middle and lower lobectomy	Recover
49 (57)	11	M	Covid-19 + DKA	20 days	Periampullary solid change	autopsy	NM	No	No	Death
50 (58)	No	M	ALL	NM	Nodular lesion	Blood and localised secretions mNGS	VCZ + L-AmB + PCZ	Yes	No	Death
51 (59)	38	M	NO	342 days	Mass	Biopsy + tissue mNGS	PCZ + AmB + Dexamethasone	No	right upper lobectomy	Recover
52 (60)	56	W	COVID-19	NM	Right-sided pyothorax	postoperative pathology	NM	No	open heart surgery	Death
53 (60)	42	M	COVID-19	NM	Cavity lesion	postoperative pathology	NM	No	lobectomy	Recover
54 (60)	72	W	COVID-19 + DM	NM	Collapse consolidation	postoperative pathology	NM	No	bilobectomy	Recover
55 (60)	67	W	COVID-19 + DM + IHD + hypertension	NM	Cavitary lung lesions and pyothorax	postoperative pathology	NM	No	open heart surgery	Recover
56 (61)	62	M	DM + hypertension + IHD	NM	Tumour-like mass necrosis	Biopsy	L-AmB + PCZ	Yes	No	Recover
57 (62)	21	W	DKA	143 days	Inflammatory mass in the right hilum	Biopsy	AmB + PCZ	Yes	total pulmonary resection	Recover
58 (63)	37	W	NO	NM	thick-walled cavity	postoperative pathology	NM	Yes	Lower lobectomy of the right lung	Recover
59 (64)	59	M	COVID-19	NM	Thick-walled cavities	sputum smear	L-AmB + AmB	No	right lower lobectomy	Recover
60 (65)	54	W	DM	78 days	nodule	BALF+ postoperative pathology	AmB + L-AmB + PCZ	Yes	lobectomy	Recover

EBUS-TBNA, endobronchial ultrasound-guided transbronchial needle aspiration; AmB, amphotericin B; BALF, bronchoalveolar lavage fluid; COVID-19, coronavirus disease 2019; CT, computed tomography; DM, diabetes mellitus; HM, hematologic malignancy; L-AmB, liposomal amphotericin B; AmBD, amphotericin B deoxycholate; ABLC, amphotericin B lipid complex; NM, not mentioned; PCZ, posaconazole; VCZ, voriconazole; AP, acute pancreatitis; DKA, Diabetic Ketoacidosis; HF, heart failure; ALL, Acute lymphoblastic leukemia; AA, Aplastic anemia; AML, Acute myeloid leukemia; SLE, Systemic lupus erythematosus; IHD, ischemic heart disease; RHS, reverse halo sign.

**Table 2:** Findings from the literature analysis.

Category	Details
Total number of cases	63 cases
Gender distribution	Male: 42 people, Female: 20 people, 1 case with gender not reported
Age	Median age is 52 years old (interquartile range [IQR], 34–60 years old)
Treatment outcomes (number of cases with outcomes: 61 cases)	Improvement: 42 cases, Death: 19 cases
Underlying diseases	DM (including diabetic ketoacidosis and diabetic nephropathy): *n* = 32 (50.8%); COVID-19: *n* = 17 (27%); Hematological oncology-related diseases: *n* = 6 (9.5%); Post-renal transplantation: *n* = 4 (6.3%); Others [Trauma: *n* = 2 (3.2%), Drug abuse, HIV, Systemic lupus erythematosus: 1 case each (1.6%)]
Chest CT findings	Typical reversed halo sign: *n* = 2 (3.2%); Crescent sign: *n* = 1 (1.6%); Mass/Consolidation: *n* = 29 (46%); Cavity: *n* = 9 (14.3%)
Diagnostic specimen types and proportions	Bronchoalveolar lavage fluid: *n* = 25 (39.7%); Tissue biopsy: *n* = 23(36.5%); Post-operative pathology: *n* = 13 (20.6%); Sputum/Blood/Pleural effusion: *n* = 11 (17.5%)
Proportion of specimens using mNGS technology	36%
Antifungal treatment (number of treated patients: 53 cases)	Death: 15 cases (28.3%), Improvement: 37 cases (69.8%), 1 case with prognosis not mentioned
Antifungal treatment combined with bronchoscopic interventional treatment (number of treated patients: 38 cases)	Death: 9 cases (23.7%), Improvement: 28 cases (73.7%), 1 case with prognosis not mentioned
Antifungal treatment combined with surgical treatment (number of treated patients: 22 cases)	Death: 2 cases (9%), Improvement: 20 cases (91%)
Antifungal treatment combined with bronchoscopy and surgical treatment (number of treated patients: 13 cases)	Death: 0 cases

## 4 Discussion and conclusion

Clinical manifestations in patients with mucormycosis lack specificity; fever, cough, and hemoptysis are common, and occasionally superficial lymph node dilatation may occur (4). In the three cases of mucormycosis of the lungs we reported, they all presented with fever, cough, sputum, and wheezing suffocation. A guideline for imaging invasive pulmonary mucormycosis recommends a chest computed tomography (CT) scan for patients with suspected lung fungal infections. The reverse halo sign and hypodensity sign are relatively typical of pulmonary mucormycosis but have a low incidence. It is also referred to as the” atoll sign,” which is characterized by an area of ground-glass clouding with a peripheral ring of solid changes. The hypointensity sign is defined as a distinct central area of low attenuation (5). Their early imaging features were unremarkable in our reported case histories, with CT often suggesting masses, solid changes, and cavities. In our review of 63 articles, mass, solid, and null forms accounted for 46.0% and 14.3% of cases, respectively, and reverse halo sign accounted for only 3.2%. Therefore, it is challenging to diagnose pulmonary mucormycosis infection based on clinical symptoms and chest computed tomography (CT) scan.

The mechanism by which mucormycosis invades the lungs is unclear. Some literature suggests that the fungus CotH7 recognizes integrin β1 as a receptor on alveolar epithelial cells, which is highly expressed in human lung tissue. This binding triggers the activation of epidermal growth factor receptor (EGFR) signaling, leading to host cell invasion and lung infection (6). In addition, fungal capsid protein homolog 3 (CotH3) interacts with glucose-regulated protein 78 (GRP78), which is produced in response to elevated blood glucose (7), and is therefore more likely to affect patients with elevated blood glucose. PM occurs mainly in immunocompromised patients, such as those with diabetes mellitus and diabetic ketoacidosis, hemato-oncology-related diseases, and organ transplantation. We reported on three patients, all of whom fit into the immunocompromised population. Among the 60 cases reported, HIV and drug abuse populations, who are equally susceptible to mucormycosis, have also been reported.

Early diagnosis and treatment can help reduce mortality in patients with mucormycosis. Mucormycosis can be identified from specimens using both culture and non-culture methods, such as tissue biopsies, bronchoalveolar lavage (BAL), or other respiratory samples, as well as serum. Fiberoptic bronchoscopy and endobronchial ultrasound-guided transbronchial needle aspiration (EBUS-TBNA) play an essential role in obtaining specimens. When specimens obtained by bronchoscopy are not diagnostic, CT-guided percutaneous biopsy should be performed. mNGS has a higher sensitivity and shorter detection cycle than traditional culture or histopathological testing for mucormycosis, making it a complementary method for early diagnosis, it can also help to detect mixed infections and provide timely notification of antimicrobial therapy, thus improving patient prognosis, and was used in 36% of specimens in the literature that we searched. However, mNGS samples are obtained from the patient’s bronchoalveolar lavage fluid, making it difficult to determine whether the microorganisms reported by mNGS are pathogens of clinical significance or simply colonizing microorganisms (8). An article demonstrates that combining histopathology and ITS sequencing is a practical approach superior to fungal culture in detecting mucormycosis in tissue-associated infections (9). In addition, different teams have developed PCR-based serodiagnostics using three quantitative polymerase chain reaction (qPCR) assays targeting *Rhizomucor* spp., *Lichtheimia* spp., *Mucor* spp., and *Rhizopus* spp., respectively (10).

The treatment of mucormycosis relies on three key elements, namely, surgery, antifungal therapy, and correction of risk factors. In this study, the term “combination therapy” refers to a multimodal treatment strategy integrating systemic antifungal agents, bronchoscopic interventions, and surgical procedures. Our literature review revealed a graded therapeutic efficacy, with triple-modality therapy demonstrating 100% efficacy, superior to antifungal-surgical dual therapy (91%), followed by antifungal-bronchoscopic combination (73.7%), and monotherapy, which showed the lowest efficacy (69.8%). A recent review (4) highlighted that multiple guidelines recommend radical surgical debridement as first-line therapy. However, another study (2) noted surgical challenges due to factors including underlying disease severity, rapid post-diagnosis mortality, and patient refusal driven by procedural risks. When surgery is contraindicated, antifungal therapy becomes critical. One study (11) identified amphotericin B as the most active antifungal agent against Mucorales. International guidelines endorse LAmB (lipid formulation amphotericin B) as first-line treatment (66). Based on our clinical experience, we employ an escalated local delivery approach for critically ill patients: intravenous LAmB combined with either nebulized amphotericin B or bronchoscopic intralesional administration. Given LAmB’s significant nephrotoxicity and hepatotoxicity, we transition to step-down therapy with isavuconazole or posaconazole after completing a full 10-day course of amphotericin B. Notably, in the included studies, combination therapy with two antifungal agents appears to improve patient survival, as supported by our Case 2 and retrieved case reports (12, 13). However, the evidence remains limited, and further studies are warranted. Finally, concurrent risk factor mitigation includes controlling blood glucose, correcting acidosis, elevating granulocyte counts, and minimizing or discontinuing glucocorticoids or immunosuppressive drugs.

## Data Availability

The original contributions presented in the study are included in the article/supplementary material, further inquiries can be directed to the corresponding author.
